# A novel transcranial MR Guided focused ultrasound method based on the ultrashort echo time skull acoustic model and phase retrieval techniques

**DOI:** 10.1038/s41598-024-62500-6

**Published:** 2024-05-24

**Authors:** Dechen Kong, Gaojie Liu, Bochao Cheng, Xu Qi, Jiayu Zhu, Qiang He, Haoyang Xing, Qiyong Gong

**Affiliations:** 1 College Of Physics, Sichuan University, Chengdu, China; 2 Huaxi MR Research Center (HMRRC), Department of Radiology, West China Hospital of Sichuan University, Chengdu, 610041 China; 3 Xiamen West China Hospital, Sichuan University, Xiamen, China; 4 Department of Radiation Oncology, Sir Run Run Shaw Hospital, Zhejiang University School of Medicine, Hangzhou, China; 5 Department of Radiology, West China Second University Hospital of Sichuan University, Chengdu, 610041 China; 6 Radiotherapy Physics & Technology Center, Cancer Center, West China Hospital of Sichuan University, Chengdu, 610041 China; 7 Central Research Institute, United Imaging Healthcare Group, Shanghai, China

**Keywords:** Translational research, Magnetic resonance imaging

## Abstract

Transcranial ultrasound stimulation (TUS) has been clinically applied as a neuromodulation tool. Particularly, the phase array ultrasound can be applied in TUS to non-invasively focus on the cortex or deep brain. However, the vital phase distortion of the ultrasound induced by the skull limits its clinical application. In the current study, we aimed to develop a hybrid method that combines the ultrashort echo time (UTE) magnetic resonance imaging (MRI) sequences with the prDeep technique to achieve focusing ventral intermediate thalamic nucleus (VIM). The time-reversal (TR) approach of the UTE numerical acoustic model of the skull combined with the prDeep algorithm was used to reduce the number of iterations. The skull acoustic model simulation therapy process was establish to valid this method’s prediction and focus performance, and the classical TR method were considered as the gold standard (GS). Our approach could restore 75% of the GS intensity in 25 iteration steps, with a superior the noise immunity. Our findings demonstrate that the phase aberration caused by the skull can be estimated using phase retrieval techniques to achieve a fast and accurate transcranial focus. The method has excellent adaptability and anti-noise capacity for satisfying complex and changeable scenarios.

## Introduction

Low-intensity ultrasound, such as transcranial ultrasound stimulation (TUS) techniques^1^, has been applied clinically for neuromodulation. Based on the fact that transcranial focused ultrasound can safely reach deep brain region associated with neurologic diseases such as essential tremors and Parkinson’s tremors^2–4^. However, a severe phase aberration arises caused by the skull^5^, while the large difference in sound velocity between the skull and brain tissue induces the significantly distorted acoustic field at the target.

Currently, numerical simulations are widely adopted in clinics to resolve the aforementioned challenges^6–11^, obtaining a patient’s skull information through computed tomography (CT) scanning, establishing a three-dimensional (3-D) skull numerical acoustic model, and using this information to estimate the phase shift or time delay of the tissue aberration and refocused beam input at the transducer^12^. This technology has been applied to tumor ablation surgery^13,14^. However, it is limited by the 3-D acoustic full simulations and may not be feasible for many real-time applications because of the computational time required^15^.

Thus, the novel focusing techniques, adaptive iterative focusing based on the acoustic radiation force impulse (ARFI) sequence^16^ were developed, called ’energy-based focusing’^17,18^. This method obtains a displacement map by inputting different coding modes used to estimate the transfer matrix (TM) of the ultrasonic propagation medium, such as Hadamard^18^, Zernike^19^, and Random^20^ et al. However, the use of these iterative algorithm to estimate the TM is time-consuming and requires a high calculation volume for improved accuracy. For example, for an array with *N* elements, at least *3N* ultrasonic sonications are required for full array calibration, which usually takes hours, leading to a temperature risk to the skin and high cumulative energy deposition. In addition, the measurement noise in ARFI images is also a key problem that influences the accuracy of this technique^21,22^.

**Figure 1:**
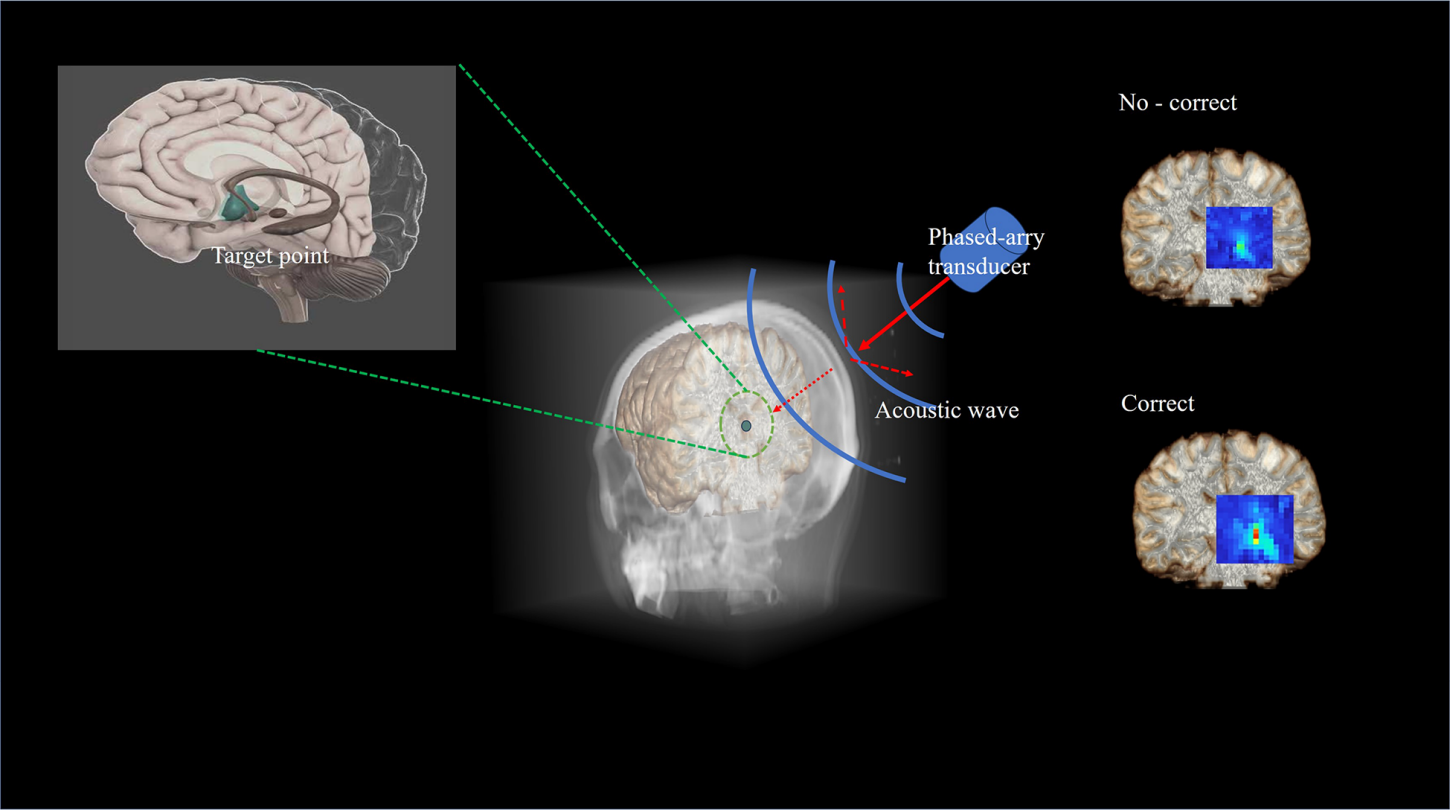
Schematic diagram of ventral intermediate thalamic nucleus (VIM) brain area stimulated by ultrasound. Due to the scattering effect of the skull on ultrasound (red line), the sub images on the right show the uncorrected and corrected focus results, respectively (blue box). The 3-D head model data is derived from CT images of a subject.

To overcome the abovementioned limitations, we utilized a transcranial focusing ultrasound hybrid technique in the current study, using ultrashort echo time (UTE) imaging to establish the initial TM as well as phase retrieval to reduce the number of ultrasonic calibrations, while maintaining adequate focusing performance in a Gaussian noise environment (see Fig. 1). This technique was validated through numerical simulations using k-Wave^23^. We hypothesis that using UTE images as the *prDeep* (a phase retrieval algorithm that is both robust and broadly applicable) initial input can drastically reduce the number of iterations required for the calibration of TUS therapy, achieving a better noise immunity and finally achieving an accurate focus.

## Results

### Prediction performances

**Figure 2:**
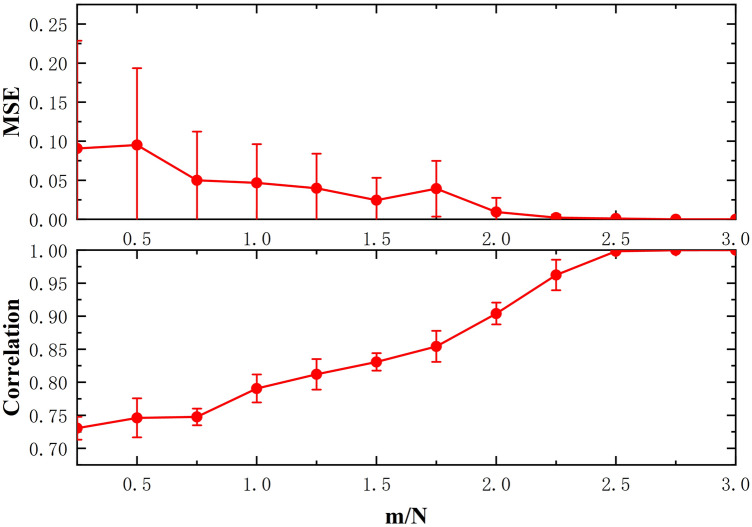
Prediction performance according to (**a**), the mean-square error (MSE) and to (**b**), the Pearson-correlation (Corr) between observation predictions using the estimated TM, and measurements of the CT modle (square root of the intensity values), as a function of the number of calibration measurements (x-axis is *m*/*N*). We repeated 10 times prDeep for three subjects to estimate the row of TM about the target point and calculated MSE and Corr, respectively. The red line represents the average of the experimental data, and the error bars denote the 95% confidence interval (CI) for increasing m/N, where m denotes the number of iterations and N denotes the elements of the transducer array ($N = 128$).

The prediction performance results of the estimated TM using the prDeep algorithm are shown in Fig. 2 . The three subjects results show that increasing *m*/*N* leads to a decrease in mean-square error (MSE) and an increase in correlation. Interestingly, we see that for $m/N > 1$, i.e., for at least one more real measurement than complex unknowns, the estimation of the TM is accurate enough to predict the observations with an average correlation of approximately 0.90 and an average MSE lower than 0.018. These results demonstrate the accuracy of the prDeep algorithm for estimating TM.

### Focusing performance

**Figure 3:**
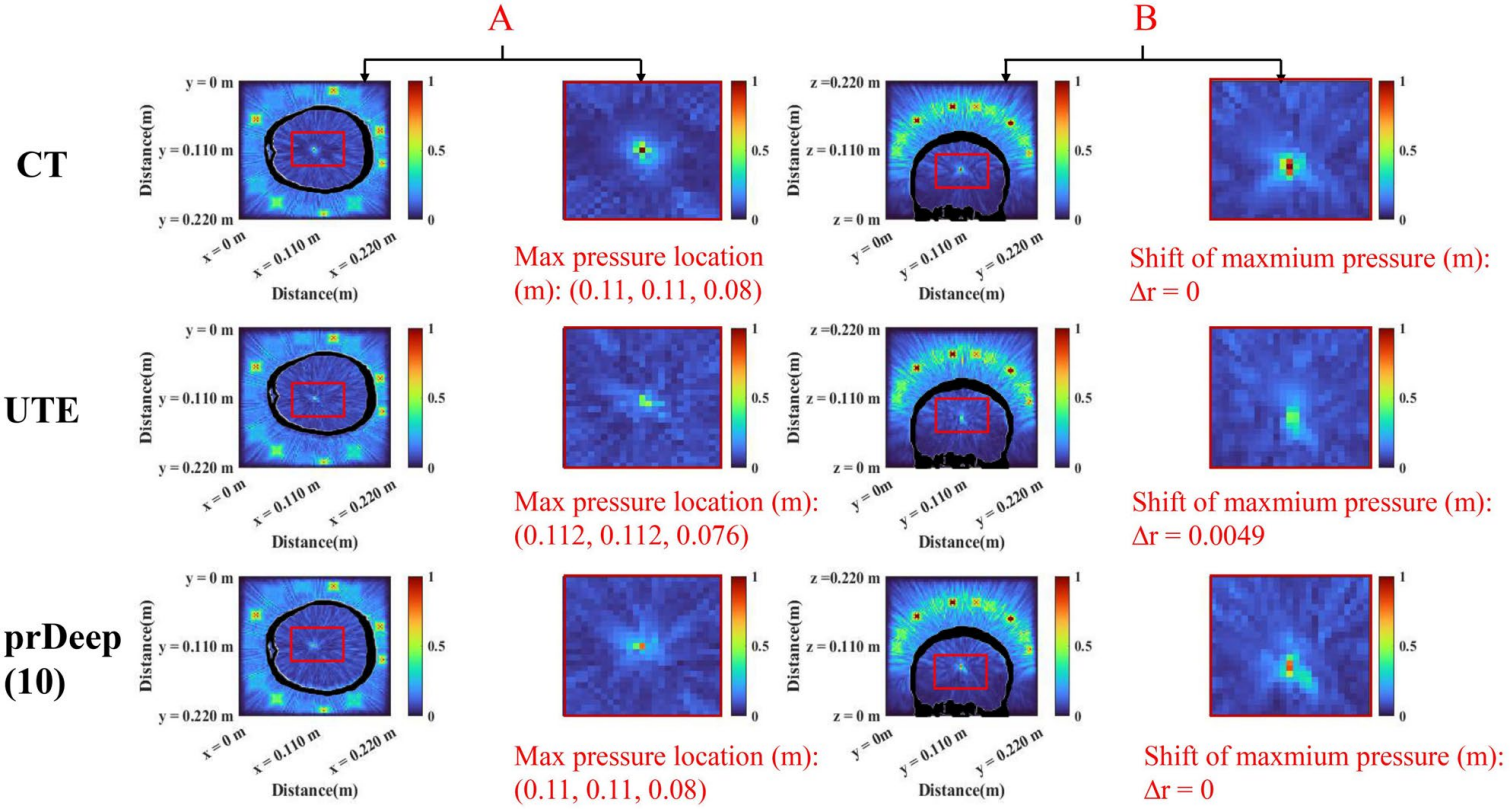
Schematic diagram of focusing results using one of the subjects as an example. (**A**), Transverse section results of acoustic simulation focal zone in different conditions include CT, UTE, and prDeep. (**B**), Coronal section results in the same way. The colorbar denotes norm sound pressure (0 to 1).

**Figure 4:**
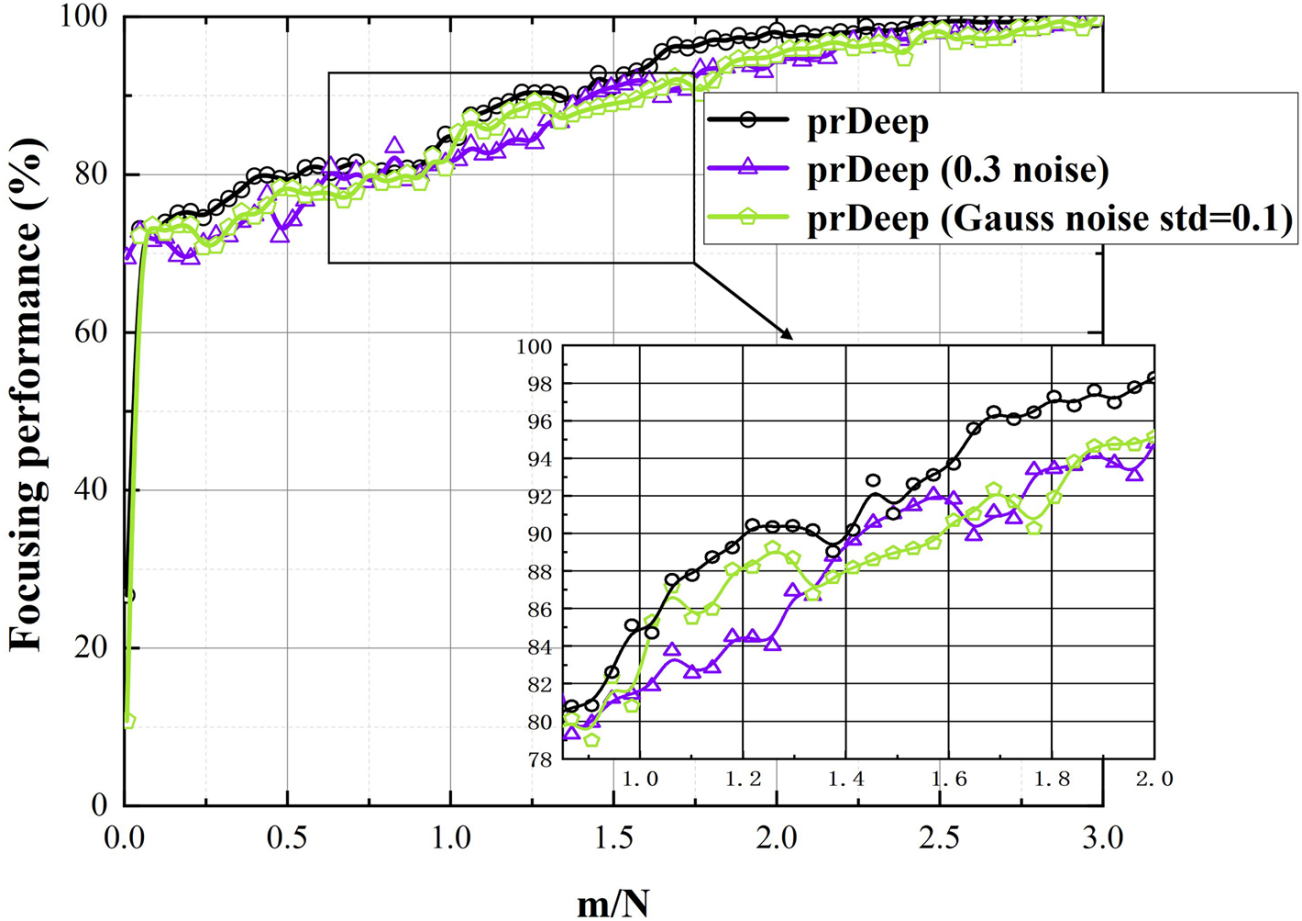
The prDeep algorithm focuses performance in different noise levels (Gaussian noise and average 0.3 times intensity experiment noise). As *m*/*N* increases, the recovery sound pressure gradually increases.

**Figure 5:**
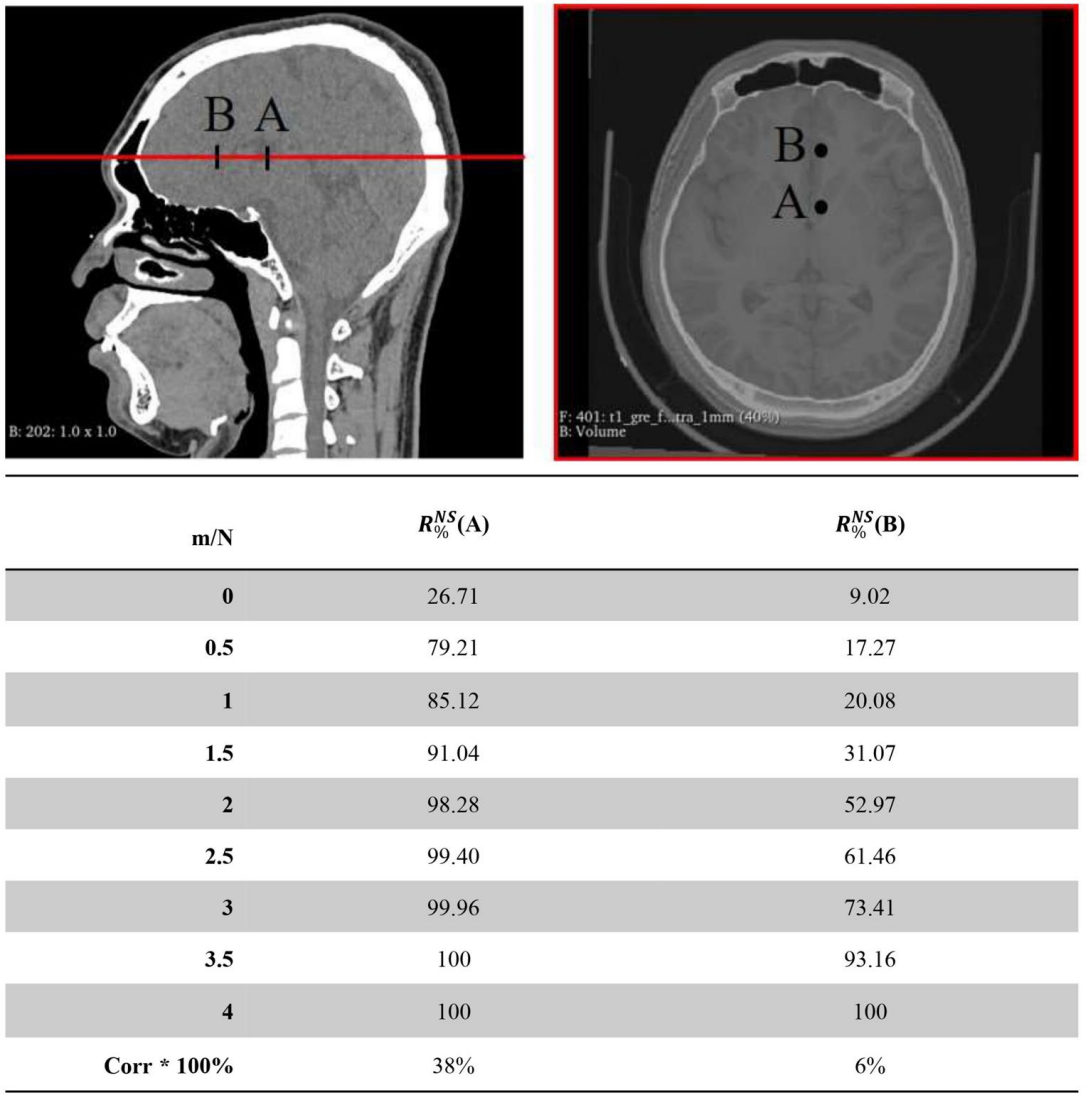
The focusing performance at different target points, with A virtually around the ventral intermediate nucleus (VIM), and B virtually around the corpus callosum, was evaluated. The CT and fusion image data used in this figure originate directly from the subject.

The simulation results that time-reversal (TR) solutions $TR_{CT}$ , $TR_{UTE}$ and $TR_{pr}$ as input to CT acoustic model displayed in Fig. 3. The calibration results of prDeep in 10 steps are shown in the last rows, where the maximum pressure is $R_{\%}^{NS} = 72\%$ and the focus shift $\Delta r = 0$ mm. The acoustic energy is concentrated in the immediate vicinity of the target, and the pressure distribution has a spherical symmetry with side lobes lower than 40% of the maximum. This demonstrates the refocusing capability of the technology. Furthermore, the prDeep technology whole focusing and noise immunity performance displayed in Fig. 4, which is the main result of this study. We find that all the considered situations yield approximately similar results whenever $m/N < 1$, regardless of whether noise is added. And, it demonstrates rapid progress in the initial 10 steps of the iteration process. This rapid initial progression is highly suitable for fast focusing, and by step 25, we can already recover 75% of the intensity. We also added the additional target. Figure 5 states that the algorithm performs at different target points. In B point, due to Corr lower, the acoustic intensity does not rapidly recover. Thus, we analyzed the relationship between initial estimation and algorithm performance, as shown in Table 1. The results indicate that when the correlation between the initial estimate and the true value of TM is less than 10% (indicating a large initial estimation bias), the algorithm needs m/N = 4 to fully recover the maximum sound intensity of the target point. Therefore, a good initial estimate can greatly reduce the number of iterations of the algorithm.

**Figure 6:**
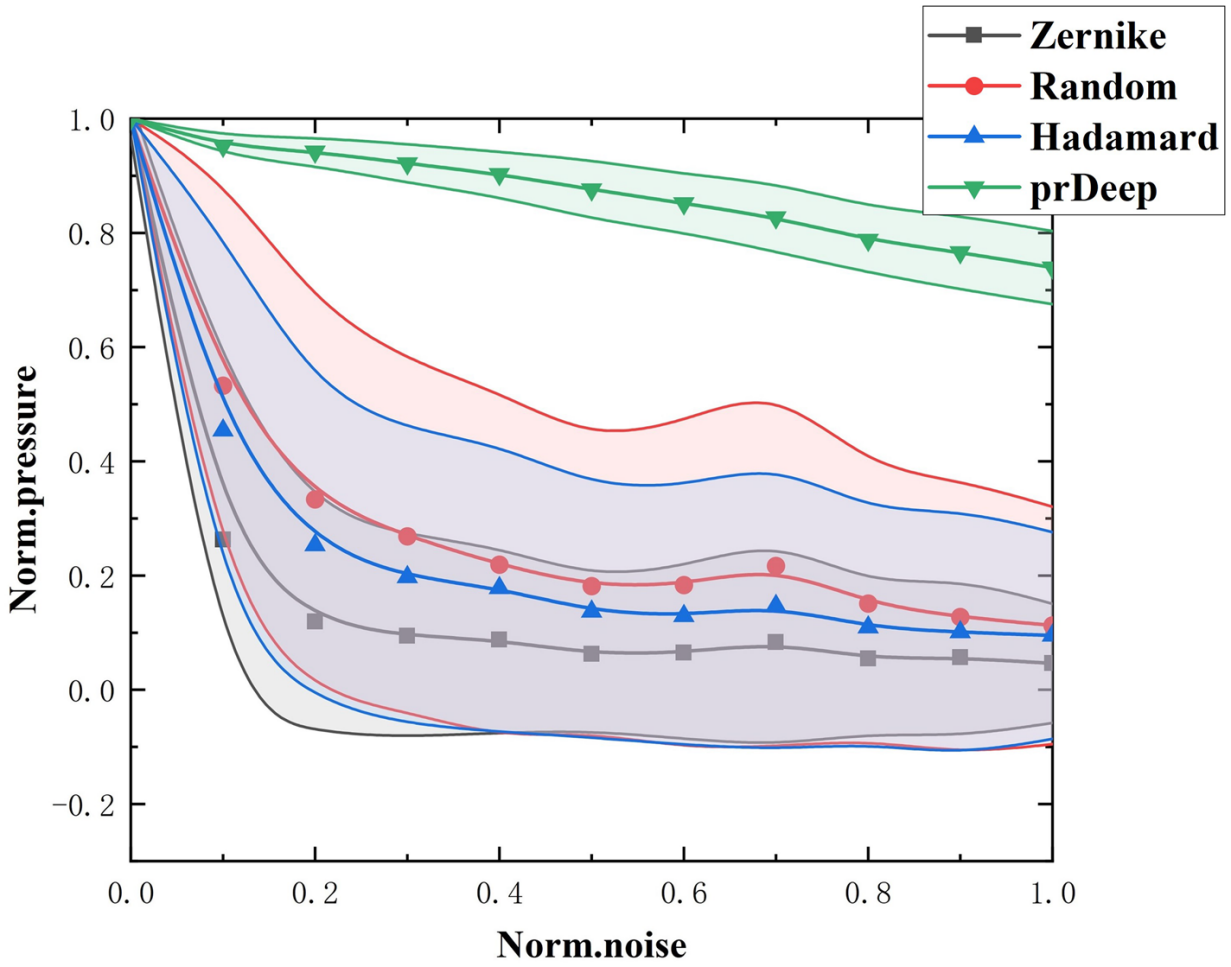
Sensitivity of the measurement process of TM of human skull to the noise level for 4 methods: normalized restored pressure at the focal point as a function of the normalized noise. Each point is a 100 computations average; solid lines are the standard deviations.

Moreover, we tested the focusing performance of the hybrid method with different standard deviations (Std = 0.1, 0.5, and 1) of Gaussian noise, as shown in Table 2 (The data for three subjects). This result indicates that this study’s proposed focused method can accurately achieve transcranial focusing within a small number ($m<N$) of iterations. To further assess the robustness and stability of this focusing technique, we compared its results with those of other methods (Hadamard, Zernike, Random, and prDeep), and all results in same simulation environment. Figure 6 shows the normalized pressure at the focal point as a function of the normalized standard deviation of noise. It is apparent that prDeep has excellent noise immunity and stability compared with the other techniques.

## Discussion

This study investigated the effectiveness of the phase retrieval (PR) algorithm and UTE imaging in TUS. We found that this hybrid method can achieve accurate transcranial ultrasound focusing, even in Gaussian noise environment and has ability of fast recovery sound pressure in the initial 10 steps.

The findings of the present study show that a combination of with UTE images and the prDeep algorithm could resolve the phase aberration challenge of the skull in transcranial ultrasound stimulation therapy. This is supported by reports that Hosseini et al.^22^ proposed using the use of a Bayesian framework to solve the phase aberration problem. On the other hand, these studies on random matrices and a digital micromirror device (DMD) adaptive focusing^24–26^ inspired our method. In practice, the limitation of the times of high iterations always obstructs the ‘energy-based focusing’ application to TUS. Thus, we referred to the random matrix calculation TM method^20^, hoping to use prior knowledge of the UTE images to reduce the number of iterations of the random matrix method^27^. The input distribution of the measurement matrix is not improved here because we expect an increase in the intensity of the target point to start from the maximum recovery intensity calculated by the UTE, rather than gradually increasing from a small initial value.

Moreover, before employing the prDeep algorithm, the time-reversed solution obtained from the UTE model can recover 68% of the acoustic intensity at target points. After 10 iterations of prDeep, the recovery of target point acoustic intensity increases to 72% and achieves a focal correction of 4.9 mm. This demonstrates the focal correction capability and the ability to recover the acoustic intensity of this method. Although our method, may not exhibit a speed advantage compared to other transcranial iterative focusing techniques^19^ (in terms of the number of iterations required to reach 90% of the gold standard intensity), it demonstrates rapid progress in the initial 10 steps of the iteration process. This rapid initial progression is highly suitable for fast focusing.

Furthermore, the measurement noise^21^ presents a significant challenge in practical applications and often hampers the development of iterative focusing techniques. We provide evidence that our method, with the incorporation of deep convolutional neural network for image denoising (DnCNN), exhibits superior noise resistance compared to other methods, making it highly applicable in practical TUS treatment scenarios. Importantly, we found that an excellent initial estimate of TM can accelerate the entire focusing process and DnCNN can ensure insensitivity to noise. Thus, in this study measurement of the TM of the UTE acoustic skull model is necessary.

Some limitations of the current study are the relatively small sample size and the fact that the DnCNN was not specially trained. This study did not use hydrophones to measure ex vivo human skull TM due to hardware limitations. In the current study, the CT acoustic modeling method and K-Wave having been validated multiple times for their accuracy^28,29^. However, the skull numerical acoustic model from the UTE images was only used to estimate TM before prDeep correction. In future work, as the number of experiments and specially trained DnCNN frameworks increase, we may gradually obtain a higher estimate of the TM efficiency and speed.

In conclusion, the current hybrid method drastically reduces the number of iterations required for TUS therapy, and achieves an accurate focus with an excellent robustness and stability to meet complex and changeable scenarios. Our findings may facilitate the application of the transcranial focused ultrasound iteration technique in actual TUS clinical therapy.

## Methods

### Theory

In practice, the total TM content is unknown and should be determined through ultrasonic sonication. Thus, when we use the TM framework to solve the phase aberration, which can standardize the focalization task as a calibration problem: “When the input matrix is given, which model is most likely to explain the observed output ?”

**Figure 7:**
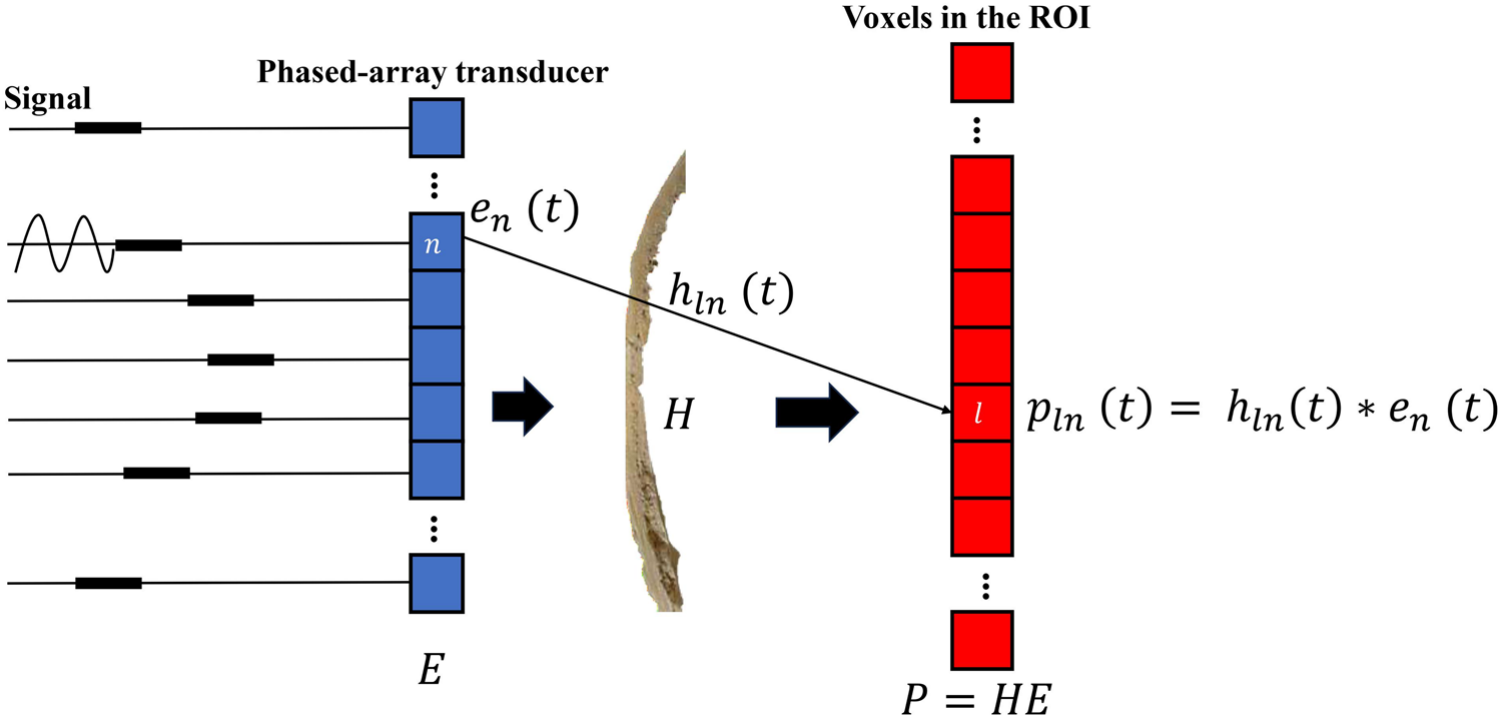
Using the TM framework describe transcranial acoustic wave propagation. When the *n*-th vibration source emits a short ultrasonic pulse $e_n(t)$, the partial observations (the square root of the local acoustic intensity) at the point of space *l* are $p_{ln}(t)$, and $h_{ln}(t)$ represents the response between the signals sent by the *n* transducer and received at the *l* point.

Formally, let *E* stand for the transducer input, which is $N \times K$ matrix. Each column of this matrix corresponds to a different input set for the transducers, in the frequency domain (amplitude and phase). The corresponding output displacements are measured, yielding the $M \times K$ matrix *P*:

1 $$\begin{aligned} P=HE \end{aligned}$$

where H is the $M \times N$ matrix, called the TM, in which each element represents the response between the input unit *n* and the target point *m*, and *H* contains the patient’s skull information and the corresponding sound field information, shown in Fig. 7. In practical work, measuring the skull TM often requires the use of needle hydrophones^15,28,30^. The transducer sequentially emits pulse signals at the same frequency, while the hydrophone records the scattered acoustic signals behind the skull. This allows extraction of their amplitude and phase at the same frequency, thereby measuring the complex values of the TM. However, placing hydrophones inside the human brain is impractical, so the above method is often used for ex vivo measurement of the skull TM. For example, Thomas et al used the needle hydrophone measurement 1345 points, and the elements of the transducer were successively triggered insonated during 500 $\upmu$s at the same electrical power (0.5 W). The signal received at the hydrophone was recorded and its amplitude and phase at 650 kHz were extracted for each element (1024) of the transducer. The TM at 650 kHz was thus recorded^15^. In addition, by adopting conjugating-transposing of the system, we obtain:

2 $$\begin{aligned} P^T=|HE|^T=|E^T H^T | \end{aligned}$$

where $.^T$ denotes the conjugate-transpose of a matrix/vector, A ’classic’ PR problem^31,32^ can be seen here. Considering the input matrix $E^T$, each element of the complex-valued column of $H^T$ is estimated using each column of $P^T$.

Additionally, according to the spatial reciprocity relation^12^ , when the temporal delta function $\delta (t)$ is applied on the *l*th space point ($F_{l0}=\left\{ 0,...,0,1,0,...0\right\}$), the signal received by the *n*th sensor is $h_{nl}(t)$. Thus, spatial reciprocity implies that the transpose of *H*, $^tH$ corresponds to the propagation matrix between the control points and the array elements. In the first step of a TR experiment, one of the space points behaves like a source^33^ :

3 $$\begin{aligned} E_0={^tH}F_{l0}=\{H\}_{l} \end{aligned}$$

where $\{H\}_{l}$ is the *l*th row of H. Furthermore, the TR can be expressed as (Time reversal means conjugation in the frequency domain):

4 $$\begin{aligned} P_{TR} =H(E_0)^*=H({^tH}F_{l0})^*=HH^TF_{l0} \end{aligned}$$

where $HH^T$ is called the time-reversal operator ($H^T=(^t{H})^*$) and * is the conjugation operation, which is described in detail in^34^. Thus, the estimated value in Eq. 2 corresponds to the solution provided by the TR approach^35^.

### PrDeep algorithm

As described in the theoretical framework in the previous subsection. The prDeep algorithm can to resolve the TM in Eq. 2, which have two main advantages:

- The prDeep was created by leveraging the regularization-by-denoising framework and a convolutional neural network denoiser,
- its generic framework, enabling its use in applications for the calibration of the TM.

prDeep includes prRED^36^ and DnCNN^37^, which have excellent noise resistance and good versatility. Regularization by denoising (RED) can be combined with any denoiser to regularize the general inverse problem. PrRED is the process of using RED to solve the PR problem. In this study, we continued to use the denoising DnCNN framework trained by Metzler^38^ without adding a new noise level. Here, we outline the main line of the prDeep algorithm and its use for TM calibration. We recommend that readers refer to the original article^38^ for an understanding of the content of this section. According to Eq. 1, let *f*(*H*) be the data fidelity term (according to Eq. 2, we set *H* to $H^T$, conjugate transpose of the TM), to calculate the distance between the estimator and the accurate measurement to ensure that the result conforms to our due process. *R*(*H*) is the standard term (penalty function) used to evaluate the recovery degree of *H* and prevent overfitting from deviating from the actual value. Therefore, we constructed following the cost function

5 $$\begin{aligned} f(H)+R(H) \end{aligned}$$

RED regularization is defined as

6 $$\begin{aligned} R(H)=\lambda H^T(H-D(H))/2 \end{aligned}$$

where D(H) refers to any denoiser in which the amplitude loss function is used

7 $$\begin{aligned} f(H)=\frac{1}{2}||P_l-{|E_{m}H|}||_2^2 \end{aligned}$$

where $E_{m}$ refers to the measurement matrix or input matrix (we apply the random matrix of +1 and -1 Bernoulli distribution); thus, there is a non-convex optimization problem

8 $$\begin{aligned} \mathop {\arg \min }\limits _{H}\frac{1}{2}||P_l-{|E_{m}H|}||_2^2+\lambda H^T(H-D(H)) \end{aligned}$$

The FASTA solver (approximate gradient method) is used to solve the above equation^39^. We define $z=|E_{m}H|$; thus, a useful sub gradient

9 $$\begin{aligned} z-P_l\circ \frac{z}{|z|}\in \partial _z\frac{1}{2}||P_l-{z}||_2^2 \end{aligned}$$

where $\circ$ denotes the Hadamard product and $\partial _ Zf(z)$ refers to the subdifferential of *f* relative to *z*.

The derivation process of the algorithm is not detailed. For further description of the algorithm and DnCNN trained, please refer to^38^. The public implementations of prDeep is available at prDeep.

### Image acquisition and material preparation

Three physically active subjects with no history of nervous system disease participated in the study. All data collection protocols were approved by the Huaxi MR Research Center Investigational Review Board. All methods were carried out in accordance with the relevant guidelines and regulations. Informed consent was obtained from all participants in the study. MR head images were obtained on a UIH 3.0T system (uMR790; Shanghai United Imaging Healthcare, Shanghai, China) using a 24-channel combined head and neck coil. Images were obtained using a 3D UTE sequence with single-echo time. Imaging parameters were as follows: TE = 0.12 ms, TR = 9 ms, TI = 21 ms, flip angle = 25$^\circ$, bandwidth = 900 Hz/px, slice thickness = 1.8 mm, and Field of View (FOV) 23 cm $\times$ 23 cm. CT images were acquired on a UIH uCT780. All the work runs on a computer with an 11th Gen Intel i7. Software: FSL, MIPAV, 3D slicer, MRIcroGL, and Matlab. The toolkits used^40,41^ were K-Wave, matconvnet, FASTA, cld-amp toolbox, prDeep.

### Acoustic model of skull

**Figure 8:**
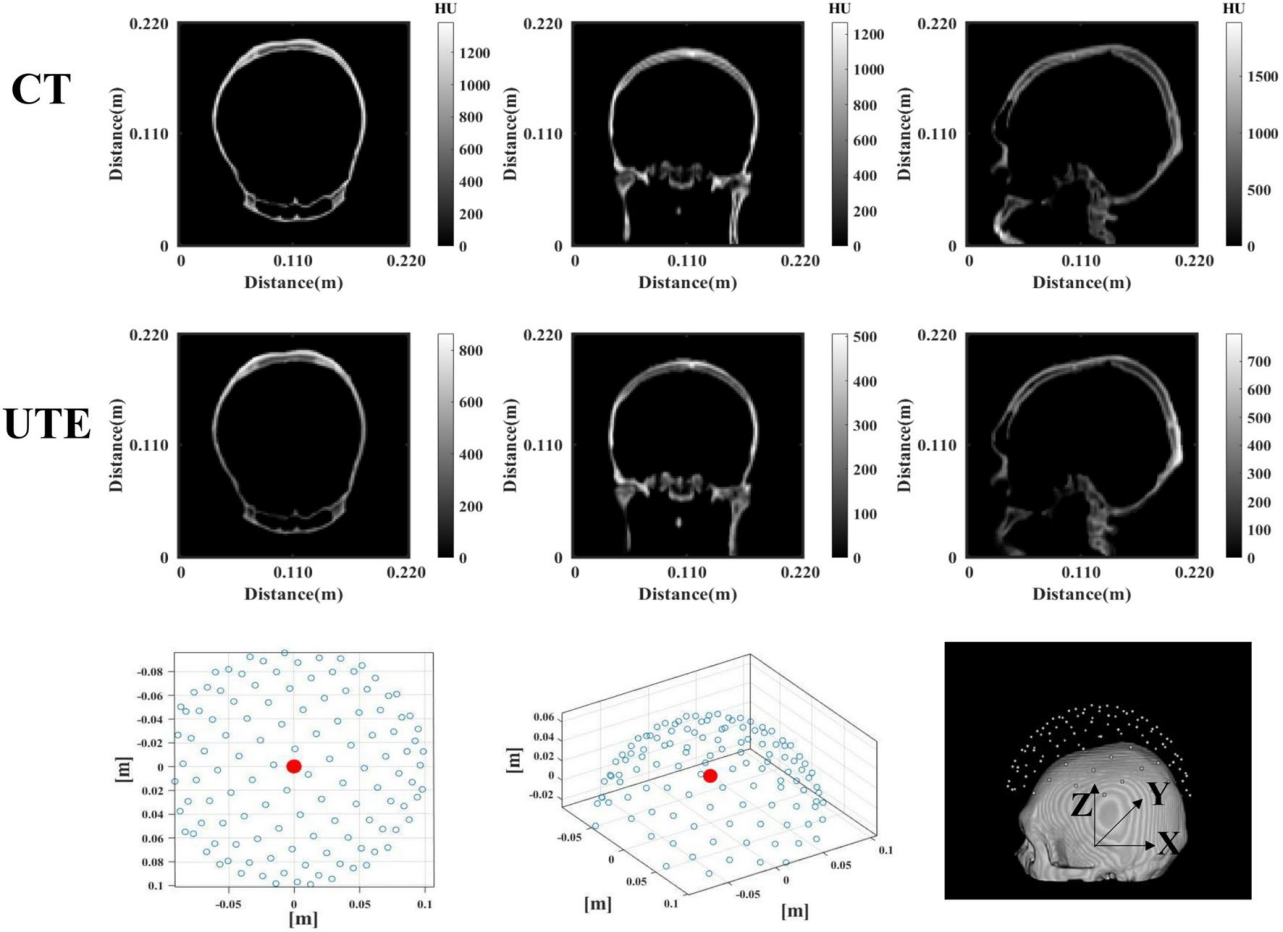
Skull segmentation result of CT and UTE images, where the colorbar denotes signal intensity. The last row displays the phased array of simulation, where the black point represents the geometric center of the array, the number of vibration sources is 128, the radius is 0.1 m. The lower right corner figure represents the numerical skull model and corresponding X, Y, and Z directions.

To establish a 3-D acoustic model of the skull, we used FSL (FLIRT)^42^ and MIPAV to finish the CT and UTE image registration and segmentation (see Fig. 8)^43^. To match the signal intensity of the CT image (unit: HU) with the gray value of the corresponding UTE image, we mapped the UTE signal intensity to the CT comparable intensity by logarithmic scaling according to the method^44^ of ( fit line: $\log (UTE) = 7.3170 \times 10^{-4}CT + 5.0211, r^2= 0.283,p < 0.05$ , and log denotes natural logarithm). Subsequently, to establish an acoustic model corresponding to the head, it is necessary to understand the acoustic characteristics of the skull, including the speed of sound^45^, density, and the acoustic attenuation coefficient^46,47^. These parameters can be estimated from the relationship between the Hounsfield units (HU) values of the CT images and the porosity. Thus, for more details and formulas regarding UTE alternatives to CT imaging in transcranial focused ultrasound, please refer to^48^.

### Simulation

In this subsection, we focus on the various set parameters of the transcranial ultrasound simulation, including the k-grid size, and voxel size et al. Furthermore, all simulations used a k-space pseudo-spectral method-based solver, the K-Wave toolbox in MATLAB^23^ .

The CT scans were from a 24-year-old adult male, the transverse plane ($512 \times 512$ pixels) had the spatial resolution of 0.47 mm, and the longitudinal spatial resolution was 1 mm. Before simulating the acoustic waves, we set voxels lower than zero HU to $HU_{\min } = 0$, corresponding to that of water. Voxels with values greater than 2400 were set to $HU_{\max }=2400$, which is the expected value for the cortical bone. Linear interpolation was performed to adjust CT scans to the desired spatial resolution. And, all acoustic parameters were derived from HU. The UTE (MRI) scans were from the same subject, the transverse plane ($256 \times 256$ pixels) had the spatial resolution of 0.89 mm, and the longitudinal spatial resolution was 1.8 mm, with the same post-processing method. The difference was that we used regression line synthetic CT. The grid was fixed at 2000 $\upmu$m (points per wavelength $PPW = 3$), corresponding to the grid dimension of $\lambda /3$, where $\lambda$ is the wavelength in water at 250 kHz, with respect to the stability criteria of the K-Wave^49^. The temporal time step ($\Delta t = 2.38 \mu s$, points per period $PPP = 16.8$) was automatically calculated using the K-Wave toolbox to respect the 0.3 Courant-Friedrich-Lewy (CFL) condition in the cortical bone. The number of time-steps was set to auto. The number of ultrasonic array elements $N = 128$, in the shape of a hemisphere with the radius of 0.1 m (The pitch of the elements is about 0.7 mm, and the size is a dot of space.), and the signal propagation of a 40 $\upmu$s toneburst of central frequency $f = 250$ kHz was simulated. The reason for choosing it is that frequency selection for nerve regulation is generally between $0.25 \sim 1$ MHz to stimulate the target area. Previous findings have shown that, at lower frequencies, the probability of motor cortex and visual neural responses is higher for any given ultrasound intensity^50^. In this study, the targeted area is located at 70 mm$\pm 5$ mm from the top of the skull (0.11, 0.11, 0.08) (unit: m) in the Cartesian coordinate system, which is virtually around the ventral intermediate thalamic nucleus (VIM). This functional region is of clinical interest in the treatment of essential tremors using TUS. We also add the additional target (0.11, 0.09, 0.08) (unit: m), which is located around the corpus callosum.

In this work, we use prDeep to solve the PR problem in Eq. 2. To validate this method, We used Eq. 1 to replace the complete transcranial ultrasound transmission simulation and calculated the corresponding recovery sound pressure *P* in the target point. Before introducing the iteration process in detail, there are the following definitions: P represents the recovered acoustic pressure at target points, E represents the input matrix of the phased-array transducer, which is adjustable ($128\times 1$ matrix), and H represents the TM of the skull, containing both skull and spatial acoustic field information. Following the TM measurement methods outlined in the theoretical section, TM was measured under CT and UTE acoustic models ($H_{CT}$ and $H_{UTE}$, $1\times 128$ matrix), with $H_{CT}$ serving as our target for TM recovery.

**Figure 9:**
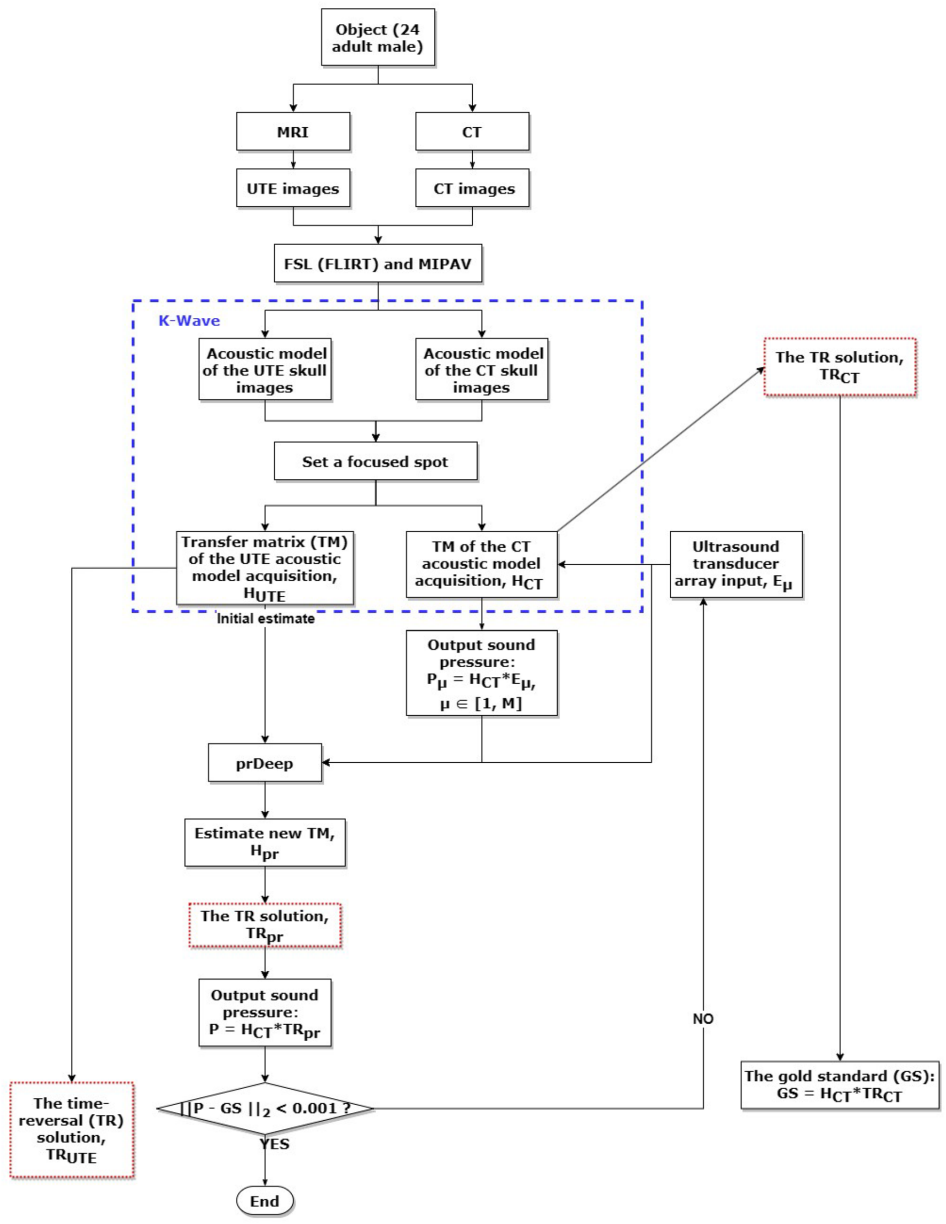
Flow chart of the simulation, where GS denotes the gold standard, P is the sound pressure of the target point and M is the maximum number of iterations. The blue dashed box denotes simulation work in the K-Wave toolbox. The red dashed box denotes resolution of TR approach.

Further details on the iterative process are as follows: Firstly, before formal iteration, the prDeep algorithm allows for the utilization of initial estimates of the TM to reduce iteration steps, with $H_{UTE}$ used here. Secondly, E serves as the input matrix of the ultrasound array, employing a Bernoulli random distribution of +1 and -1 equal probabilities, eliminating the need to consider specific spatial positions and shapes of ultrasound sources as in the Zernike method^19^. Based on $P=H_{CT}*E$, the corresponding target point acoustic pressure P is obtained under different ultrasound inputs. Inputting E and P into prDeep yields estimates of the TM. Finally, based on Eq. 4, $TR_{pr}$ is obtained, representing the TR solution at target points, which is then substituted into $P=H_{CT}*TR_{pr}$ to recover acoustic pressure and determine its proximity to the gold standard (GS). If sufficiently close, iteration ceases; otherwise, new E and corresponding P are inputted. The detailed iterative steps are illustrated in Fig. 9.

### Sensitivity of the hybrid techniques to the noise level

Gaussian random noise was added numerically. The standard deviation noise of the Gaussian distribution varied from 0 (no additional noise) to 1. Subsequently, for each noise level value, the phase correction and maximum pressure restored at the focal point were computed using different methods (Hadamard, Zernike, Random, and prDeep). This enabled us to assess the effectiveness and robustness of the proposed method.

### Data analysis methods

In this study, we used two methods to assess the focused methods: prediction and focusing performance. First, the prediction performance of the prDeep algorithm estimated TM was evaluated according to the mean-square error (MSE) and Pearson correlation (Corr) between the moduli of the prDeep predicted measurements and the actual observed measurements.

10 $$\begin{aligned} MSE= &  \frac{||y-|\hat{H}^{estimate}E|||_2^2}{||y||_2^2} \end{aligned}$$

11 $$\begin{aligned} Corr= &  \frac{cov(\hat{H}^{estimate}, \hat{H}^{stand})}{\sigma {\hat{H}^{estimate}} \sigma {\hat{H}^{stand}}} \end{aligned}$$

where $\hat{H}^{estimate}$ denotes hybrid method estimated result, and $\hat{H}^{stand}$ denotes the TM of the calculation in the skull acoustic model from CT images. $\sigma$ denotes the standard deviation.

Finally, we obtained the TR solution of TM from CT, UTE acoustic model, and prDeep algorithm iteration results: $TR_{CT}$, $TR_{UTE}$ and $TR_{pr}$ (indicated by the red dashed box in Fig. 9). These TR solutions were used as transducer array inputs for CT acoustic model to assess the focusing effect. In addition, to assess the maximum pressure restored in the simulation results at the target point, we computed the ratio $R^{NS}_\%$ between the maxpressure of the simulation and the GS^12^ maximum pressure $GS = H_{CT}\times TR_{CT}$.

12 $$\begin{aligned} R^{NS}_\% = \frac{ P^{Max pressure}_{sim}}{GS} \times 100 \end{aligned}$$

To assess the focus shift of the simulated transcranial focused ultrasound,

13 $$\begin{aligned} \Delta r = r_{GS} - r_{simu} \end{aligned}$$

where $\Delta r$ denotes a feature used to evaluate how close to the target the energy is actually localized.

**Table 1:** The relationship between initial estimation and algorithm performance.

Corr * 100%	95.07%	84.23%	73.27%	63.28%	51.84%	47.02%	35.68%	21.01%	< 10%
m/N									
0	97.01	44.39	71.22	29.32	36.82	16.83	12.78	27.10	9.02
0.5	97.42	85.46	80.07	65.84	61.67	54.31	44.52	34.33	17.27
1	98.75	89.31	87.72	74.1	62.4	65.58	51.36	37.65	20.08
1.5	99.54	92.5	90.38	79.39	70.69	76.38	54.50	38.89	31.07
2	100	97.38	95.32	93.3	87.72	84.22	68.14	49.70	52.97
2.5	100	98.22	98.25	92.02	92.54	95.36	75.84	62.25	61.46
3	100	100	100	98.32	99.29	99.11	99.46	82.05	73.41
3.5	100	100	100	100	100	100	100	95.5	93.16
4	100	100	100	100	100	100	100	100	100

**Table 2:** The partial data of focusing performance of prDeep algorithm in different noise levels (each data is from three subjects and computation average and standard deviation).

*m*/*N*	$R_{\%}^{NS}$(No-noise)	$R_{\%}^{NS}$(Gaussian noise, $std=0.1$)	$R_{\%}^{NS}$(Gaussian noise, $std=0.5$)	$R_{\%}^{NS}$(Gaussian noise, $std=1$)
0	$34.98\pm 32.11$	$29.53\pm 25.43$	$3.78\pm 0.82$	$25.63\pm 20.70$
0.5	$74.78\pm 4.10$	$76.26\pm 1.92$	$72.10\pm 0.90$	$73.51\pm 2.25$
1	$80.89\pm 3.66$	$82.75\pm 2.29$	$73.11\pm 3.12$	$75.88\pm 1.61$
1.5	$89.91\pm 5.90$	$86.93\pm 4.66$	$83.17\pm 2.65$	$77.64\pm 5.08$
2	$98.85\pm 0.91$	$96.18\pm 1.44$	$89.08\pm 0.95$	$84.20\pm 0.65$
2.5	$99.73\pm 0.30$	$98.89\pm 0.54$	$92.28\pm 1.06$	$86.62\pm 1.75$
3	$99.98\pm 0.02$	$99.99\pm 0.01$	$95.93\pm 0.09$	$89.99\pm 0.52$

## Data Availability

The data that support the findings of this study are available upon reasonable request from the corresponding author.
